# The distal C terminus of the dihydropyridine receptor β_1a_ subunit is essential for tetrad formation in skeletal muscle

**DOI:** 10.1073/pnas.2201136119

**Published:** 2022-05-04

**Authors:** Anamika Dayal, Stefano Perni, Clara Franzini-Armstrong, Kurt G. Beam, Manfred Grabner

**Affiliations:** ^a^Department of Pharmacology, Medical University of Innsbruck, A-6020 Innsbruck, Austria;; ^b^Department of Physiology and Biophysics, Anschutz Medical Campus, University of Colorado, Aurora, CO 80045;; ^c^Department of Cell and Developmental Biology, University of Pennsylvania School of Medicine, Philadelphia, PA 19104

**Keywords:** excitation–contraction coupling, skeletal muscle, tetrad formation, voltage-gated Ca^2+^ channel, β subunit

## Abstract

The skeletal muscle dihydropyridine receptor (DHPR) β_1a_ subunit is indispensable for full trafficking of DHPRs into triadic junctions (i.e., the close apposition of transverse tubules and sarcoplasmic reticulum [SR]), facilitation of DHPRα_1S_ voltage sensing, and arrangement of DHPRs into tetrads as a consequence of their interaction with ryanodine receptor (RyR1) homotetramers. These three features are obligatory for skeletal muscle excitation–contraction (EC) coupling. Previously, we showed that all four vertebrate β isoforms (β_1_–β_4_) facilitate α_1S_ triad targeting and, except for β_3_, fully enable DHPRα_1S_ voltage sensing [Dayal *et al.*, Proc. Natl. Acad. Sci. U.S.A. 110, 7488–7493 (2013)]. Consequently, β_3_ failed to restore EC coupling despite the fact that both β_3_ and β_1a_ restore tetrads. Thus, all β-subunits are able to restore triad targeting, but only β_1a_ restores both tetrads and proper DHPR–RyR1 coupling [Dayal *et al.*, *Proc. Natl. Acad. Sci. U.S.A.* 110, 7488–7493 (2013)]. To investigate the molecular region(s) of β_1a_ responsible for the tetradic arrangement of DHPRs and thus DHPR–RyR1 coupling, we expressed loss- and gain-of-function chimeras between β_1a_ and β_4_, with systematically swapped domains in zebrafish strain *relaxed* (β_1_-null) for patch clamp, cytoplasmic Ca^2+^ transients, motility, and freeze-fracture electron microscopy. β_1a_/β_4_ chimeras with either N terminus, SH3, HOOK, or GK domain derived from β_4_ showed complete restoration of SR Ca^2+^ release. However, chimera β_1a_/β_4_(C) with β_4_ C terminus produced significantly reduced cytoplasmic Ca^2+^ transients. Conversely, gain-of-function chimera β_4_/β_1a_(C) with β_1a_ C terminus completely restored cytoplasmic Ca^2+^ transients, DHPR tetrads, and motility. Furthermore, we found that the nonconserved, distal C terminus of β_1a_ plays a pivotal role in reconstitution of DHPR tetrads and thus allosteric DHPR–RyR1 interaction, essential for skeletal muscle EC coupling.

## Significance

Vertebrate skeletal muscle excitation–contraction coupling (ECC) is based on Ca^2+^-influx–independent interchannel cross-talk between DHPR and RyR1. The skeletal muscle DHPR complex consists of the main, voltage-sensing, and pore-forming α_1S_ subunit, the auxiliary β_1a_, α_2_δ-1, γ_1_ subunits, and Stac3. The DHPRβ_1a_ subunit plays an essential role in full triad targeting of DHPRα_1S_, voltage sensing, and tetrad formation (grouping of four DHPRs)—the three prerequisites for skeletal muscle ECC. Hence, a lack of DHPRβ_1a_ results in a lethal phenotype in both β_1_-null mice and zebrafish. Here, we identified the nonconserved, distal C terminus of DHPRβ_1a_ as playing a pivotal role in the formation of DHPR tetrads, and thus allosteric DHPR–RyR1 coupling, essential for proper skeletal muscle ECC.

Excitation–contraction (EC) coupling in skeletal muscle is initiated by depolarization of the muscle cell membrane induced by motor neuron input, which subsequently induces myofibril contractions. This transduction event depends on junctions between the surface membrane and its invaginations (transverse [T] tubules) and the sarcoplasmic reticulum (SR), in structures termed Ca^2+^ release units. The dihydropyridine receptor (DHPR) in the T-tubular membrane of the muscle cell functions as voltage sensor for this excitation signal. EC coupling in vertebrate skeletal muscle is based on Ca^2+^-influx–independent interchannel protein–protein interaction between the DHPR and ryanodine receptor (RyR1) in the SR membrane (1–3). Because of this physical interaction, the depolarization-induced conformational change of the DHPR is transmitted to the RyR1 channel, which opens to release large amounts of Ca^2+^ ions from the SR Ca^2+^ stores—a process that is the final trigger for myofibril contraction (4, 5).

The skeletal muscle DHPR complex consists of the central, pore-forming, and voltage-sensing α_1S_ subunit and the accessory subunits β_1a_, α_2_δ-1, and γ_1_ (6–8). Among them, the α_1S_ and the β_1a_ subunits are indispensable for skeletal muscle EC coupling (9–11). Akin to DHPRα_1S_-null (*dysgenic*) (9) and RyR1-null (*dyspedic*) (12) mice, β_1_-null mice (10) and β_1_-null zebrafish (strain *relaxed*) (11) show a lethal phenotype due to complete absence of skeletal muscle contractility that leads to asphyxia. Besides the two canonical DHPR subunits, the junctional proteins Stac3 and junctophilin-2 (JP2) are also crucial for proper DHPR–RyR1 interaction that enables concerted voltage-induced SR Ca^2+^ release in skeletal muscle (13).

In DHPRβ_1_-null zebrafish strain *relaxed*, a lack of the β_1a_ subunit results in 1) reduced DHPRα_1S_ expression in the T-tubular membrane, 2) elimination of α_1S_ charge movement, and 3) a lack of the arrangement of DHPRs into groups of four (tetrads) opposite every other RyR1 (11). These three features are prerequisite for the tight protein–protein interaction between the DHPR and RyR1 and thus form the structural–functional basis for skeletal muscle EC coupling. Using zebrafish strain *relaxed* as a very convenient expression system, we previously showed that all four vertebrate β-isoforms (β_1_–β_4_), and also the ancestral β-subunit of *Musca domestica* (β_Μ_) (14), are able to fully target α_1S_ into triads (15). Additionally, except for β_3_, all other vertebrate β-isoforms are able to restore full charge movement (16) (*SI Appendix*, Fig. S1). Consequently, despite the surprising fact that β_3_, akin to β_1a_, is able to accurately cause the organization of DHPRs into tetrads, it is unable to restore EC coupling (16). Interestingly, only expression of β_1a_ fulfills all the three structural–functional prerequisites, i.e., proper DHPR triad and tetrad restoration, as well as proper charge movement facilitation and consequently, accurate DHPR–RyR1 interaction (15). As a result, native skeletal muscle β_1a_ is the only DHPRβ subunit that supports proper skeletal muscle EC coupling (*SI Appendix*, Fig. S1).

To identify a structural domain(s) of β_1a_ essential for restoration of DHPR voltage sensing, and hence to probe how the DHPRα_1S_–β_1a_ interaction affects this initial step of EC coupling, we previously performed reconstitution studies in the *relaxed* system using chimeras between β_1a_ and β_3_ (16). Voltage-gated Ca^2+^ channel β-subunits are intracellular proteins with a five-domain organization and two conserved domains, the *src* homology 3 (SH3) and guanylate kinase (GK) domains that are connected by the variable HOOK region and flanked by variable N and C termini (17–20). The outcome of systematic domain swapping between β_1a_ and β_3_ in the study of Dayal et al. (16) revealed a pivotal role of the β_1a_ SH3 domain and the C terminus in charge movement restoration. The results indicate that this domain–domain interaction is dependent on a SH3-binding polyproline (PXXP) motif in the proximal C terminus of the β_1a_ subunit. Consequently, it was concluded that the β_1a_ subunit, apparently via its SH3–C-terminal PXXP interaction, adopts a discrete conformation required for inducing a proper conformational change in the α_1S_ subunit crucial for “turning on” its voltage-sensing function (16).

Nevertheless, we are just beginning to understand the importance of distinct molecular domains of the β_1a_ subunit in skeletal muscle EC coupling. In the present study, we characterized the second crucial structural prerequisite, tetrad formation, which contrary to the promiscuous structural property of DHPR triad targeting by all β-subunits, is shared by only β_1a_ and β_3_ (*SI Appendix*, Fig. S1). To identify β_1a_ domains responsible for proper DHPR tetrad formation and thus proper DHPR–RyR1 protein–protein interaction as a basis for induction of SR Ca^2+^ release and finally muscle contractility/motility, we expressed putative loss- and gain-of-function chimeras with systematically swapped domains between β_1a_ and β_4_ in zebrafish strain *relaxed* for patch clamp, cytoplasmic Ca^2+^ transients, motility, and freeze-fracture electron microscopy (EM) analyses.

Here we report that our loss- and gain-of-function chimeras indicate the importance and exclusivity of the nonconserved distal C terminus of β_1a_ in DHPR tetrad formation and thus a DHPR–RyR1 interaction essential for proper skeletal muscle EC coupling. Within the distal C terminus, we found that a hydrophobic surface (L_496_L_500_W_503_), previously postulated to be important for activation of RyR1 (21), does not appear to play a role in EC coupling Ca^2+^ release. Based on these results, we propose a model in which the distal β_1a_ C terminus enables a conformation of the β-subunit, which in turn causes the intracellular domains of α_1S_ to assume the positioning required for the interaction with RyR1 and thus the tetradic arrangement of DHPRs (22, 23).

## Results and Discussion

### The β_4_ Subunit Is the Apt Isoform for Mapping the Domain(s) of β_1a_ Crucial for Tetrad Formation in Skeletal Muscle.

To elucidate the importance of distinct β_1a_ domain(s) for DHPR tetrad formation the first step was to identify the most apt β-isoform, which lacked this property and thus could serve as a molecular tool for chimerization with β_1a_. Proper DHPR tetrad formation is a common attribute of β_1a_ and β_3_, but is missing in β_2a_ (15, 16). Although not directly tested, we postulated that β_4_ might also be poor at restoring DHPR tetrads because it only restored ∼50% of cytoplasmic Ca^2+^ transients upon expression in *relaxed* myotubes despite its ability to completely support DHPR triad targeting and charge movement (16). β_4_ was given preference over β_2a_ as a chimerization partner with β_1a_, because it is phylogenetically older than β_2a_ (16) and thus has overall lower amino acid homology to β_1a_ than β_2a_ (60.1% compared to 65.7%, respectively), which also holds true for the aligned C termini (24.2% versus 31.8%, respectively). Equally important, when exploring the role of the C terminus in charge movement restoration (16), we saw that β_4_ does not have as long a C terminus as β_2a_ (117 versus 193 residues, respectively).

**Fig. 1.**
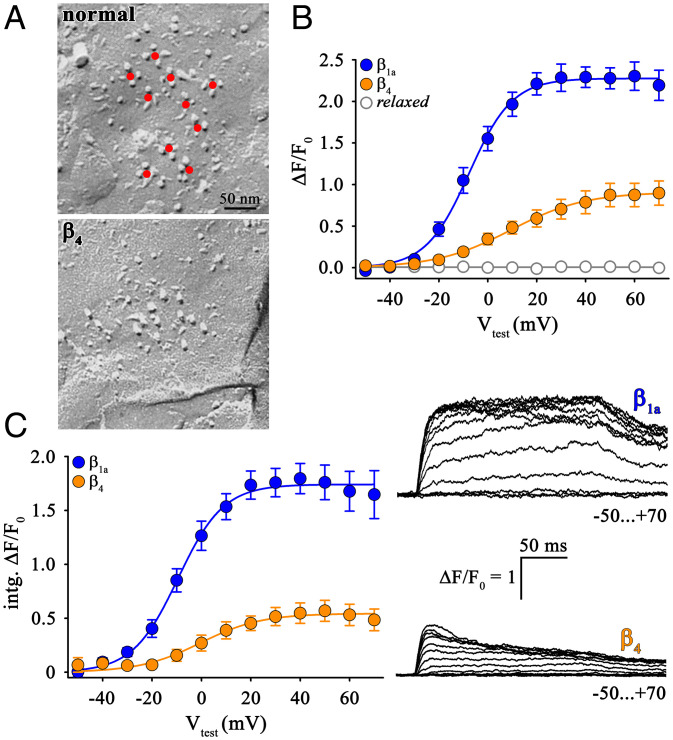
Absence of DHPR tetrad restoration in β_4_-expressing *relaxed* myotubes. (*A*) Freeze-fracture replicas of peripheral couplings in tail myotomes of 27- to 30-hpf zebrafish. Control myotomes (*Top*) show arrangement of DHPR particles in tetrads (center indicated by red dots), organized in orthogonal arrays. In β_4_-expressing *relaxed* zebrafish (*Bottom*) DHPR tetrads show a lack of tetrad formation. (Scale bar, 50 nm.) (*B*) Quantification of voltage dependence of cytoplasmic Ca^2+^ transients yielded (Δ*F/F*_0_)_max_ values that are significantly lower (*P* < 0.001) in β_4_ (*n* = 13)- compared to β_1a_ (*n* = 9)-expressing *relaxed* myotubes. Δ*F/F*_0_ values recorded from untransfected *relaxed* myotubes were below detection level (*n* = 10). (*C*) Similarly, plots of voltage dependence of the integral of the Δ*F/F*_0_ transients in response to 200-ms test depolarizations indicate a highly significant difference (*P* < 0.001) in the total amount of Ca^2+^ released between *relaxed* myotubes expressing β_1a_ (*n* = 9) or β_4_ (*n* = 12) subunit. (*Right*) Representative Δ*F/F*_0_ recordings from *relaxed* myotubes expressing β_1a_ or β_4_. (Scale bars, 50 ms [horizontal], Δ*F/F*_0_ = 1 [vertical].) Error bars indicate SEM. *P* determined by unpaired Student’s *t* test.

As we postulated, DHPR tetrads were not detectable in β_4_-expressing *relaxed* myotubes (Fig. 1*A*), making β_4_ useful for mapping the molecular domain(s) of β_1a_ essential for tetrad formation via chimeric constructs. In β_4_-expressing *relaxed* myotubes, cytoplasmic Ca^2+^ transients [(Δ*F/F*_0_)_max_ = 1.02 ± 0.15, *n* = 13] was significantly larger (*P* < 0.001) than in untransfected *relaxed* myotubes (below detection limit [bdl], *n* = 10) but significantly smaller when compared (*P* < 0.001) to β_1a_ [(Δ*F/F*_0_)_max_ = 2.30 ± 0.15, *n* = 9] (Fig. 1*B*). Moreover, Δ*F/F*_0_ in the β_4_-expressing myotubes had a voltage dependence (Fig. 1*B*) that was >12 mV rightwardly shifted compared to β_1a_ (*V*_1/2_: β_4_, 6.05 ± 3.42 mV, *n* = 13; β_1a_, −6.87 ± 2.65 mV, *n* = 9; *P* < 0.01). The transients also had a different time course in β_1a_- and β_4_-expressing myotubes. In the β_1a_-expressing myotubes, the transients had a steep rise followed by a plateau during the 200-ms depolarization (Fig. 1 *C*, *Upper Right*), which presumably represents a rapid, transient release of Ca^2+^ into the cytoplasm followed by a lower, sustained release just sufficient to balance the Ca^2+^ removal mechanisms (24, 25). In the β_4_-expressing myotubes both the transient and sustained release appear to be reduced so that the initial rise is smaller and that the transient decays during the pulse because the sustained release is outweighed by the removal processes (Fig. 1 *C*, *Lower Right*). To obtain a signal related roughly to total release (transient plus sustained), we integrated (intg.) the transients and plotted the area versus test potential, which also revealed a significant difference (*P* < 0.001) between β_4_ and β_1a_ (intg.Δ*F/F*_0_: β_4_, 0.64 ± 0.10, *n* = 12; β_1a_, 1.97 ± 0.18, *n* = 9; Fig. 1*C*).

### The C Terminus of the β_1a_ Subunit Is Key for Proper DHPR–RyR1 Coupling.

**Fig. 2.**
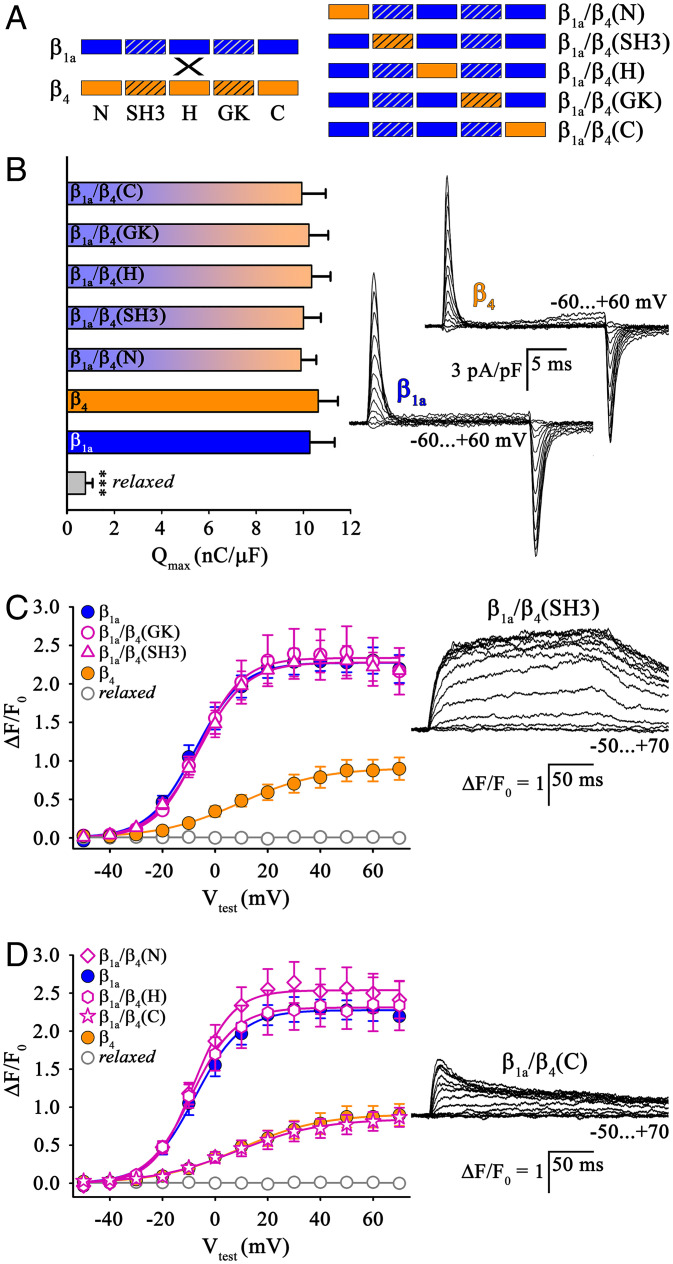
Loss-of-function β_1a_/β_4_ chimeras revealed the importance of the β_1a_ C terminus in skeletal muscle DHPR–RyR1 coupling. (*A*) Block schemes of domain organization of putative loss-of-function β_1a_/β_4_ chimeras with systematic exchange of N terminus (N), SH3 domain (SH3), HOOK region (H), GK domain (GK), or C terminus (C) of β_1a_ (blue) by β_4_ sequences (orange). Homologous SH3 and GK domains are represented by hatched boxes. (*B*, *Left*) Analyses of voltage dependence of integrated outward gating currents normalized to cell capacitance exhibited maximum charge movement (*Q*_max_) values indistinguishable (*P* > 0.05) between *relaxed* myotubes expressing β_1a_ (*n* = 16), β_4_ (*n* = 12), β_1a_/β_4_(N) (*n* = 21), β_1a_/β_4_(SH3) (*n* = 19), β_1a_/β_4_(H) (*n* = 24), β_1a_/β_4_(GK) (*n* = 15), or β_1a_/β_4_(C) (*n* = 13). *Q*_max_ values from untransfected *relaxed* myotubes were slightly above detection level (*P* < 0.001, *n* = 11). (*Right*) Representative *Q* recordings from *relaxed* myotubes expressing either β_1a_ or β_4_. (Scale bars, 5 ms [horizontal], 3 pA/pF [vertical].) (*C* and *D*) Cytoplasmic Ca^2+^ transient restoration was comparable (*P* > 0.05) between *relaxed* myotubes expressing β_1a_ (*n* = 9), β_1a_/β_4_(SH3) (*n* = 17), β_1a_/β_4_(GK) (*n* = 11), β_1a_/β_4_(N) (*n* = 14), or β_1a_/β_4_(H) (*n* = 16). By contrast, Δ*F/F*_0_ values were significantly lower (*P* < 0.001) for chimera β_1a_/β_4_(C) (*n* = 12) and similar (*P* > 0.05) to those of β_4_ (*n* = 13). Exemplar Ca^2+^ transient recordings from *relaxed* myotubes expressing β_1a_/β_4_(SH3) (*C, Right*) or β_1a_/β_4_(C) (*D, Right*). (Scale bars, 50 ms [horizontal], Δ*F*/*F*_0_ = 1 [vertical].) Error bars indicate SEM. *P* determined by unpaired Student’s *t* test, ***P < 0.001.

To explore the role of the β_1a_ domain(s) in tetrad formation, we constructed a set of β_1a_/β_4_ chimeras in which the N terminus (N), SH3 domain (SH3), HOOK region (H), GK domain (GK), and C terminus (C) of β_1a_ were systematically swapped with corresponding β_4_ sequences (Fig. 2*A*). To test whether all the β_1a_/β_4_ chimeras were functionally expressed in *relaxed* myotubes, we measured DHPRα_1S_ outward (on) charge movement (*Q*_on_). The *Q*_max_ values displayed by all β_1a_/β_4_ chimeras (*n* = 13 to 24) were not significantly different (*P* > 0.05) from the basis constructs β_1a_ (10.28 ± 1.07 nC/μF; *n* = 16) and β_4_ (10.63 ± 0.85 nC/μF; *n* = 12) (Fig. 2*B*).

β_1a_/β_4_ chimeras with SH3 and GK domains derived from β_4_ completely restored cytoplasmic Ca^2+^ transients in *relaxed* myotubes [(Δ*F/F*_0_)_max_: 2.28 ± 0.19, *n* = 17 and 2.36 ± 0.32, *n* = 11, respectively] to the level of β_1a_ (*P* > 0.05) (Fig. 2 *C*, *Left*). Profiles of these cytoplasmic Ca^2+^ transients exhibited kinetics typical for β_1a_ with a sustained plateau (Fig. 2 *C*, *Right*), indicating normal DHPR–RyR1 interaction. Chimeras in which either the nonconserved N terminus or HOOK region of β_1a_ was replaced by corresponding β_4_ sequences also displayed restoration of Δ*F/F*_0_ [(Δ*F/F*_0_)_max_: β_1a_/β_4_(N), 2.53 ± 0.24, *n* = 14; β_1a_/β_4_(H), 2.29 ± 0.25, *n* = 16], not significantly different (*P* > 0.05) from β_1a_ control myotubes (Fig. 2*D*). Notably, in contrast to the other constructs, chimera β_1a_/β_4_(C), carrying the nonconserved C terminus of β_4_ restored Ca^2+^ transients [(Δ*F/F*_0_)_max_ 0.88 ± 0.13, *n* = 12] that did not differ significantly from β_4_ (*P* > 0.05) but were significantly (*P* < 0.001) smaller than those of β_1a_ (Fig. 2*D*). The results above emphasize the importance of the β_1a_ C terminus in proper DHPR–RyR1 coupling.

### The Greater Length of the β_4_ C Terminus Is Not Responsible for the Impairment of DHPR–RyR1 Coupling.

**Fig. 3.**
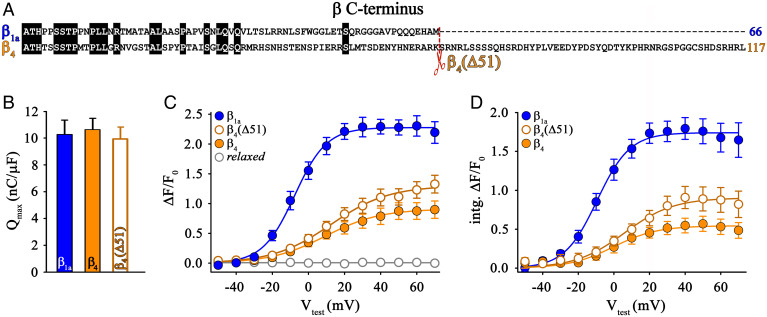
Length of the β_4_ C terminus is not crucial for skeletal muscle DHPR–RyR1coupling. (*A*) Amino acid sequence alignment depicting variable lengths of the C termini of β_1a_ and β_4_ subunits (GenBank accession nos.: rabbit β_1a_, M25514; rat β_4_, L02315). To determine whether the length of the C terminus was functionally critical, the last 51 amino acids from the β_4_ C terminus were deleted, yielding mutant β_4_(Δ51). (*B*) *Q*_max_ values were indistinguishable (*P* > 0.05) between *relaxed* myotubes expressing the deletion mutant β_4_(Δ51) (*n* = 17), β_1a_ (*n* = 16), or β_4_ (*n* = 12). (*C*) Maximal Ca^2+^ transients (Δ*F/F*_0_)_max_ for β_4_(Δ51) expressing *relaxed* myotubes (*n* = 10) was similar (*P* > 0.05) to that of β_4_ (*n* = 13). (*D*) Similarly, total maximal Ca^2+^ transients (intg.(Δ*F/F*_0_)_max_) for β_4_(Δ51) expressing *relaxed* myotubes was statistically indistinguishable (*P* > 0.05) from that of β_4_. Error bars indicate SEM. *P* determined by unpaired Student’s *t* test.

Since the C terminus of β_4_ is markedly longer (117 residues) than that of β_1a_ (66 residues) (Fig. 3*A*), the question arose whether the difference in length between the two isoforms is responsible for the significant difference in cytoplasmic Ca^2+^ transient restoration (Fig. 1 *B* and *C*). Consequently, we removed the distal 51 residues from the β_4_ C terminus to yield construct β_4_(Δ51). Full restoration of charge movement (*Q*_max_: 9.94 ± 0.91, *n* = 17) upon expression of β_4_(Δ51) in *relaxed* myotubes demonstrated that the expression of the deletion mutant did not significantly (*P* > 0.05) differ from β_1a_ and β_4_ (*Q*_max_: 10.28 ± 1.07, *n* = 16 and 10.63 ± 0.85, *n* = 12, respectively) (Fig. 3*B*). Nonetheless, peak Ca^2+^ transients for the mutant β_4_(Δ51) were significantly (*P* < 0.001) smaller than for β_1a_ [(Δ*F/F*_0_)_max_ of 1.30 ± 0.14, *n* = 10 compared to 2.30 ± 0.15, *n* = 9 for β_1a_] and not significantly different (*P* > 0.05) from β_4_ (1.02 ± 0.15, *n* = 13) (Fig. 3*C*). The same is true after comparing the integral of the Ca^2+^ transients during the 200-ms test pulses. Maximal intg.Δ*F/F*_0_ for truncation mutant β_4_(Δ51) was comparable (*P* > 0.05) to β_4_ (0.92 ± 0.12, *n* = 10 and 0.64 ± 0.10, *n* = 12, respectively), and significantly smaller (*P* < 0.001) than for β_1a_ (intg.Δ*F/F*_0_ of 1.97 ± 0.18, *n* = 9) (Fig. 3*D*). Thus, the greater length of the C terminus of β_4_ does not appear to be responsible for impairing DHPR–RyR1 coupling.

### The Distal C Terminus of β_1a_ Is Crucial for the Functional and Structural Interactions between DHPRs and RyR1.

**Fig. 4.**
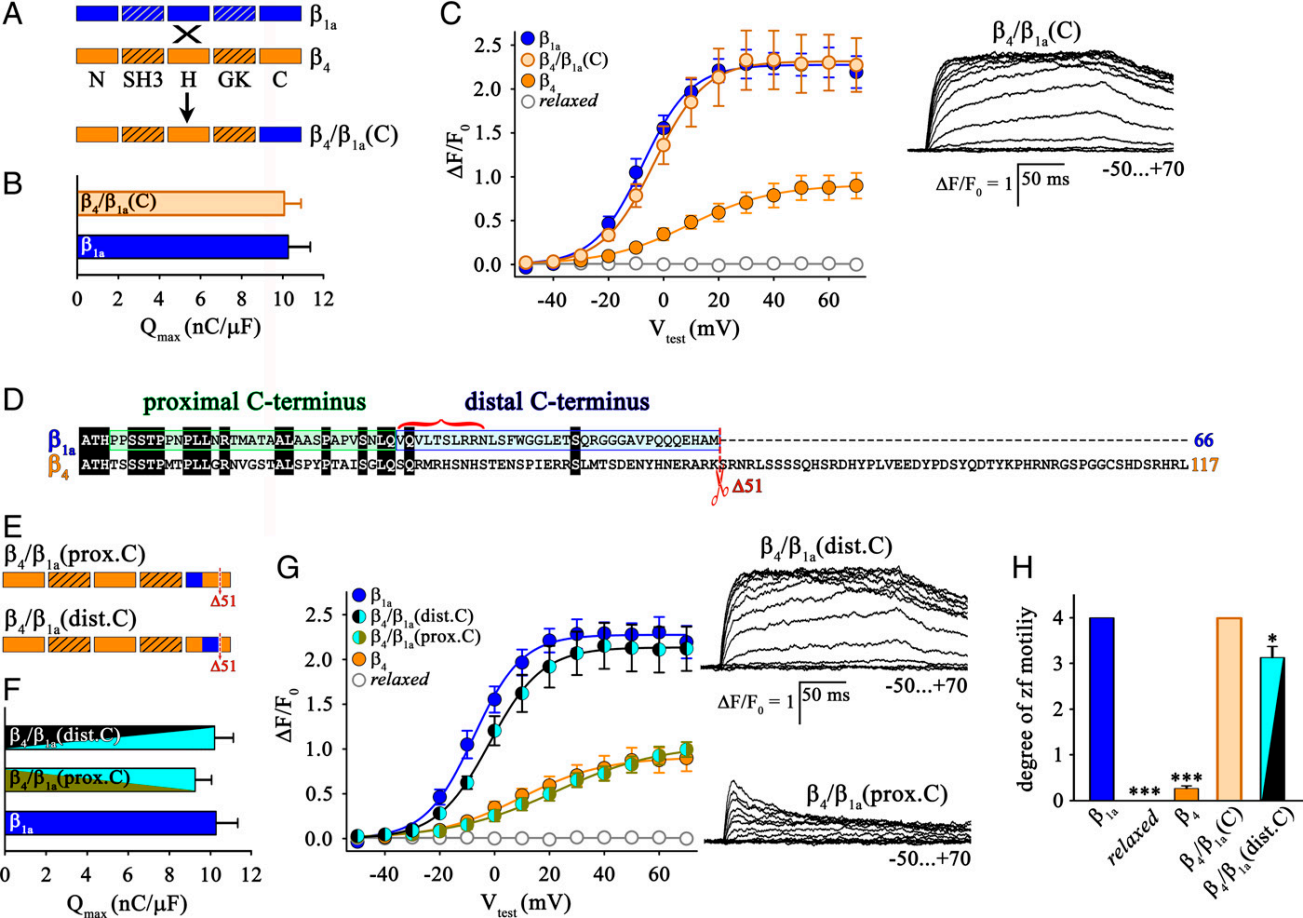
The distal C terminus of β_1a_ is crucial for skeletal muscle EC coupling. (*A*) Block scheme of domain organization of gain-of-function chimera β_4_/β_1a_(C), where the the C terminus of β_4_ (orange) was replaced by a corresponding β_1a_ sequence (blue). (*B*) *Q*_max_ values in *relaxed* myotubes expressing either chimera β_4_/β_1a_(C) (*n* = 18) or β_1a_ (*n* = 16) were comparable (*P* > 0.05). (*C*) Quantification of voltage dependence of cytoplasmic Ca^2+^ transients yielded significantly higher (*P* < 0.001) (Δ*F/F*_0_)_max_ values for chimera β_4_/β_1a_(C) (*n* = 12) compared to β_4_ (*n* = 13) but indistinguishable (*P* > 0.05) from that of β_1a_ (*n* = 9) expressing *relaxed* myotubes. (*Right*) Exemplar cytoplasmic Ca^2+^ transient recordings from *relaxed* myotubes expressing chimera β_4_/β_1a_(C). (Scale bars, 50 ms [horizontal], Δ*F/F*_0_ = 1 [vertical].) (*D*) Amino acid sequence alignment of C termini of β_1a_ and β_4_ depicting the homologous proximal C terminus (green box) and heterologous distal C terminus (blue box). Red bracket indicates the highly homologous sequence in the distal C terminus of β_1a_ revealed from sequence alignments of β_1a_ from several vertebrate species (fish to mammals) (*SI Appendix*, Fig. S2*B*). (*E*) Block scheme of domain organization of chimeras β_4_/β_1a_(prox.C) and β_4_/β_1a_(dist.C), where the proximal and distal C terminus of β_4_ (orange) were exchanged by corresponding β_1a_ sequences (blue). (*F*) *Q*_max_ values were indistinguishable (*P* > 0.05) between *relaxed* myotubes expressing chimera β_4_/β_1a_(prox.C) (*n* = 11), β_4_/β_1a_(dist.C) (*n* = 19), or β_1a_ (*n* = 16). (*G*) Quantification of voltage dependence of cytoplasmic Ca^2+^ transients yielded (Δ*F/F*_0_)_max_ values that were significantly lower (*P* < 0.001) for chimera β_4_/β_1a_(prox.C) (*n* = 15)- compared to β_1a_ (*n* = 9)-expressing *relaxed* myotubes. However, *relaxed* myotubes expressing chimera β_4_/β_1a_(dist.C) (*n* = 14) exhibited pronounced Ca^2+^ transients, equivalent (*P* > 0.05) to β_1a_ transfected myotubes (*n* = 13). (*Right*) Exemplar Ca^2+^ transient recordings from *relaxed* myotubes expressing chimera β_4_/β_1a_(dist.C) or β_4_/β_1a_(prox.C). (Scale bars, 50 ms [horizontal], Δ*F/F*_0_ = 1 [vertical].) (*H*) Quantification of spontaneous or touch-evoked coiling of 27- to 30-hpf *relaxed* zebrafish injected with β_1a_ (*n* = 35), β_4_ (*n* = 202), β_4_/β_1a_(C) (*n* = 79), and β_4_/β_1a_(dist.C) (*n* = 58) mRNA. Degree of motility was indistinguishable (*P* > 0.05) between *relaxed* zebrafish expressing β_4_/β_1a_(C) or β_1a_. *Relaxed* zebrafish expressing β_4_/β_1a_(dist.C) displayed robust spontaneous coiling only slightly lower (*P* = 0.02) than β_1a_. Conversely, β_4_-injected *relaxed* zebrafish showed either no (*n* = 151) or very weak (*n* = 51) coiling following tactile stimulation and thus, highly significantly lower motility compared to (*P* < 0.001) β_1a_-expressing *relaxed* zebrafish. Uninjected *relaxed* zebrafish displayed neither spontaneous nor tactile-induced motility (*P* < 0.001, *n* = 28). Error bars indicate SEM. *P* determined by unpaired Student’s *t* test, *P < 0.05; ***P < 0.001.

We next constructed and tested a mirror chimera to the loss-of-function chimera β_1a_/β_4_(C), namely chimera β_4_/β_1a_(C), where the β_4_ C terminus was exchanged with the corresponding β_1a_ sequence (Fig. 4*A*). *Relaxed* myotubes expressing chimera β_4_/β_1a_(C) showed functional DHPR membrane expression as indicated by full restoration of charge movement comparable (*P* > 0.05) to β_1a_ (*Q*_max_: 10.07 ± 0.83, *n* = 18 and 10.28 ± 1.07, *n* = 16, respectively) (Fig. 4*B*). Moreover, *relaxed* myotubes expressing chimera β_4_/β_1a_(C) exhibited Ca^2+^ transient levels ((Δ*F/F*_0_)_max_: 2.34 ± 0.32, *n* = 12) that were significantly larger (*P* < 0.001) than those of β_4_ (1.02 ± 0.15, *n* = 13) and comparable (*P* > 0.05) to β_1a_ [(Δ*F/F*_0_)_max_: 2.30 ± 0.15, *n* = 9] (Fig. 4*C*). As a guide for identifying the regions of the β_1a_ C terminus most important for interaction with RyR1, we aligned the C termini of β_1a_ and β_4_, which reveals 45% overall homology in the proximal C terminus and only 6% in the overlapping region of the distal C terminus (Fig. 4*D*). Although divergent from β_4_, the distal C terminus of β_1a_ shows an overall homology of 34% among various phylogenetically diverse vertebrates (*SI Appendix*, Fig. S2), including complete identity of the initial 10 residues (indicated by the red bracket in Fig. 4*D*). Thus, we hypothesized that the distal β_1a_ C terminus (dist.C) would have a stronger impact on EC coupling than the proximal C terminus (prox.C). To test this hypothesis, we constructed chimera β_4_/β_1a_(prox.C), containing the first 31 C-terminal amino acid residues of β_1a_ (459 to 489), and chimera β_4_/β_1a_(dist.C), carrying the subsequent 35 C-terminal residues of β_1a_ (490 to 524) in an otherwise β_4_ sequence background (Fig. 4*E*).

Upon transfection in *relaxed* myotubes, chimeras β_4_/β_1a_(prox.C) and β_4_/β_1a_(dist.C) were equivalent in their ability to support full membrane expression of functional DHPRs as indicated by full charge movement restoration (*Q*_max_: 9.25 ± 0.81, *n* = 12 and 10.21 ± 0.92, *n* = 19, respectively) comparable (*P* > 0.05) to β_1a_ (*Q*_max_: 10.28 ± 1.07, *n* = 16) (Fig. 4*F*). However, chimera β_4_/β_1a_(prox.C) did not restore Ca^2+^ transients above the β_4_ level [(Δ*F/F*_0_)_max_: 1.06 ± 0.10, *n* = 15 and 1.02 ± 0.15, *n* = 13, respectively; *P* > 0.05] (Fig. 4*G*). In contrast to the proximal C-terminal construct, chimera β_4_/β_1a_(dist.C) led to complete restoration of cytoplasmic Ca^2+^ transients comparable (*P* > 0.05) to β_1a_ [(Δ*F/F*_0_)_max_: 2.17 ± 0.25, *n* = 14 and 2.30 ± 0.15, *n* = 9, respectively] (Fig. 4*G*). Furthermore, we performed motility tests on 27- to 30-h postfertilization (hpf) whole zebrafish. In zebrafish expressing β_1a_, the degree of motility was high, whereas in those expressing β_4_ it was only marginally greater than in the *relaxed* zebrafish (Fig. 4*H*). Also congruent to the Ca^2+^ transient data (Fig. 4*C*), the degree of motility restored was indistinguishable between *relaxed* zebrafish expressing β_1a_ and chimera β_4_/β_1a_(C) (both 4.00, *n* = 35 and *n* = 79, respectively) (Fig. 4*H*). Moreover, chimera β_4_/β_1a_(dist.C) resulted in a high extent of zebrafish motility (3.13 ± 0.24, *n* = 79), nearly (*P* = 0.02) reaching β_1a_ and β_4_/β_1a_(C) levels, but highly significantly (*P* < 0.001) above the very marginal β_4_-induced motility (0.26 ± 0.06, *n* = 202) (Fig. 4*H*).

**Fig. 5.**
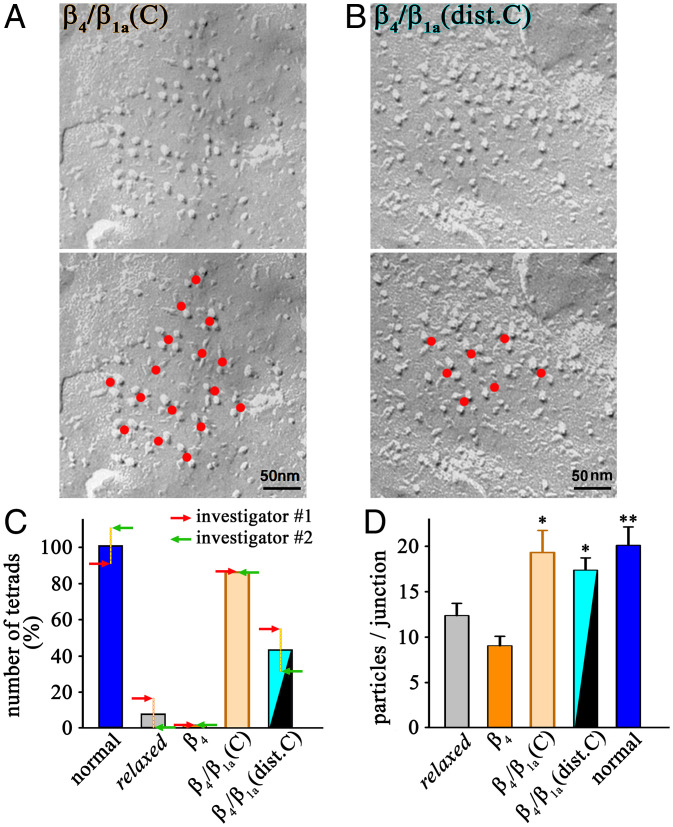
The distal C terminus of β_1a_ is crucial for DHPR tetrad formation. (*A* and *B*) Representative freeze-fracture replicas from tail muscle tissue of 27- to 30-hpf *relaxed* zebrafish expressing β_4_/β_1a_(C) (*A*) or β_4_/β_1a_(dist.C) (*B*) reveal accurate arrangement of DHPR particles in tetrads. The red dots (*Bottom*) indicate the centers of three- or four-particle tetrads and additional particles that are in the expected position for an orthogonal array. (Scale bar, 50 nm.) (*C*) Numbers of tetrads (three or four particles) determined by two independent investigators from 95 anonymized freeze-fracture images acquired from zebrafish tails, either normal controls (normal), uninjected (*relaxed*), or injected with β_4_, β_4_/β_1a_(C), or β_4_/β_1a_(dist.C) mRNA. Each bar represents mean of the counts normalized to normal zebrafish (where the mean of the two investigators’ counts was defined as 100%) and the two arrows (red and green) depict the counts of the two individual investigators (*SI Appendix*, Table S2). (*D*) Counts of DHPR particles per junction from zebrafish tails, either uninjected (*relaxed*), injected with β_4_, β_4_/β_1a_(C), or β_4_/β_1a_(dist.C) mRNA, or normal controls (normal). Error bars indicate SEM. *P* determined by unpaired Student’s *t* test, *P < 0.05; **P < 0.01.

After determining that both β_4_/β_1a_(C) and β_4_/β_1a_(dist.C) restored EC coupling Ca^2+^ transients that differed little from that in muscles of wild-type (WT) animals, we next assessed their ability to cause the tetradic organization of DHPRs. We found that tetrads were present in *relaxed* myotubes expressing either β_4_/β_1a_(C) (Fig. 5*A*) and β_4_/β_1a_(dist.C) (Fig. 5*B*), in contrast to the absence of tetrads in *relaxed* myotubes expressing β_4_ (Fig. 1 *A, Lower*). For a more quantitative comparison, unidentified images were provided to two investigators who counted the number of tetrads that were complete (four particles) or nearly complete (three particles). They were able to identify almost no tetrads in myotubes expressing β_4_, but found that tetrads were present in myotubes expressing β_4_/β_1a_(C) at levels only slightly lower than in myotubes from normal animals (Fig. 5*C*). They detected tetrads in myotubes expressing β_4_/β_1a_(dist.C) at levels about half those of normal myotubes but still substantially above those of myotubes expressing β_4_ (Fig. 5*C*).

A count by one of the two investigators of the average number of DHPR-like particles per putative junction in unidentified images (Fig. 5*D*) indicated that the accumulation of DHPRs in the junctions of muscles expressing β_4_ is comparable (*P* > 0.05) to what was observed in uninjected *relaxed* zebrafish (9.05 ± 1.04 and 12.37 ± 1.35 particles/junction, *n* = 18, respectively). At the other end of the spectrum, β_4_/β_1a_(C) expressing zebrafish show a similar number (*P* > 0.05) of DHPR-like particles per junction to that found in normal zebrafish muscles (19.32 ± 2.43 and 20.12 ± 2.03 particles/junction, *n* = 18, respectively). In the case of β_4_/β_1a_(dist.C), the clustering of DHPR-like particles in putative junctions (17.37 ± 1.36 particles/junction, *n* = 18) was substantially higher (*P* < 0.01) than that of uninjected *relaxed* zebrafish and β_4_-injected zebrafish, and comparable (*P* > 0.05) to what was measured in normal zebrafish tail muscles. These data suggest that the C-terminal domain of β_1a_ substantially contributes to increase the efficiency of DHPR junctional targeting, a contribution that is dependent on its distal part. Since junctional particle density was earlier shown to be independent of the fact of whether DHPR particles are organized in tetrads or not (11), the observed differences in particle counts per junction mirrors the differences in sizes of the junctions.

### The Hydrophobic Surface Motif (L_496_L_500_W_503_) in the Distal β_1a_ C Terminus Is Not Essential for EC Coupling.

**Fig. 6.**
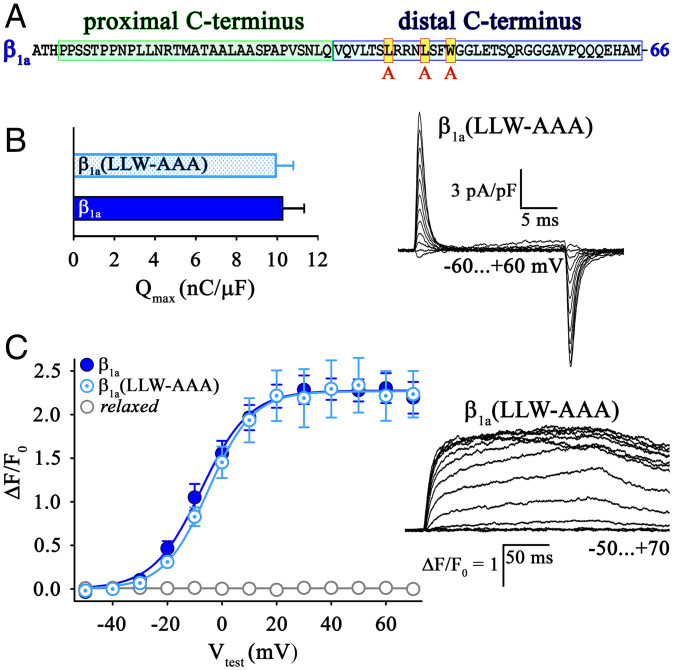
Hydrophobic residues (L_496_L_500_W_503_) in the β_1a_ distal C terminus are not important for skeletal muscle EC coupling. (*A*) Amino acid sequence of rabbit β_1a_ C terminus depicting the position of the three hydrophobic residues LLW (red box with yellow filling), which were exchanged with alanines (AAA). (*B*) *Relaxed* myotubes expressing triple mutant β_1a_(LLW-AAA) (*n* = 16) displayed *Q*_max_ values similar (*P* > 0.05) to β_1a_ (*n* = 16). (*Right*) Exemplar charge movement recording from *relaxed* myotubes expressing β_1a_(LLW-AAA). (Scale bars, 5 ms [horizontal], 3 pA/pF [vertical].) (*C*) Plots of voltage dependence of maximal Ca^2+^ transients were indistinguishable (*P* > 0.05) between β_1a_(LLW-AAA) (*n* = 13) and β_1a_ (*n* = 9)-expressing *relaxed* myotubes. (*Right*) Exemplar Ca^2+^ transient recordings from *relaxed* myotubes expressing mutant β_1a_(LLW-AAA). (Scale bars, 50 ms [horizontal], Δ*F/F*_0_ = 1 [vertical].) Error bars indicate SEM. *P* determined by unpaired Student’s *t* test.

The results described so far demonstrate that the distal C terminus of β_1a_ plays a critical role in the physical interactions between the DHPR and RyR1, which are responsible for cytoplasmic Ca^2+^ transients and tetrad formation. As to why this might be, one possibility is that the β_1a_ C terminus adopts a structure specifically suited for this role. Unfortunately, the structure of the β_1a_ C terminus has not been resolved in the cryo-EM studies (26). However, the predicted secondary structures of the distal C termini of β_1a_ and β_4_ are very similar (*SI Appendix*, Fig. S3) despite low sequence homology. Even with an overall similar structure, a more limited motif within the distal C terminus β_1a_ could be of importance. One candidate for such a role is a hydrophobic surface identified in previous work from other laboratories. In particular, using NMR spectroscopy, affinity chromatography, and RyR1 single-channel recordings in lipid bilayers, Karunasekara et al. (21) showed that a peptide corresponding to the distal 35 residues of the β_1a_ C terminus adopted a nascent α-helix, in which three hydrophobic residues (L_496_L_500_W_503_) (Fig. 6*A*) align to form a hydrophobic surface that binds to isolated RyR1 with high affinity and increases its channel activity. This effect declined significantly upon substitution of the hydrophobic residues by alanines, a swap that did not destroy the α-helical structure (21). In a follow-up study of Hernández-Ochoa et al. (27), application of a peptide corresponding to the truncated β_1a_ C terminus (V_490_–A_508_), which contained the hydrophobic LLW motif, caused a similar increase of RyR1 channel activity in lipid bilayers. Perfusion of this 19-residue peptide into murine adult skeletal muscle fibers significantly increased cytoplasmic Ca^2+^ transients, which was not observed with a scrambled control peptide. Consequently, the authors of both the studies concluded that the hydrophobic motif L_496_L_500_W_503_ is critical for EC coupling.

To test the importance of the LLW motif, we generated the mutant construct β_1a_ (LLW-AAA), in which the LLW motif was ablated by substitution with alanines (Fig. 6*A*) and expressed it in zebrafish *relaxed* myotubes. Whole-cell patch-clamp recordings revealed that charge movement restored by the mutant construct β_1a_(LLW-AAA) (*Q_max_*: 9.93 ± 0.89 nC/μF, *n* = 16) was not distinguishable (*P* > 0.05) from that restored by β_1a_ (10.28 ± 1.07 nC/μF, *n* = 16) (Fig. 6*B*), indicating that the triple alanine substitution did not affect the membrane expression of functional DHPRs. Moreover, there were no significant differences (*P* > 0.05) in cytoplasmic Ca^2+^ transients (Fig. 6*C*) between *relaxed* myotubes expressing β_1a_(LLW-AAA) or β_1a_ with respect to either magnitude [(Δ*F/F*_0_)_max_ of 2.31 ± 0.27, *n* = 13 and 2.30 ± 0.15, *n* = 9, respectively] or voltage dependence (*V*_1/2_ of −3.46 ± 1.82 mV, *n* = 13, and −6.87 ± 2.65 mV, *n* = 9, respectively). Thus, in contrast to the isolated, freely floating peptides (21, 27), the substitution of alanines for the LLW motif had no detectable effect on cytoplasmic Ca^2+^ transients when introduced into full-length β_1a_ expressed as part of the DHPR complex in intact muscle cells. Therefore, our data provide strong evidence that the L_496_L_500_W_503_ motif in the distal C terminus of the DHPRβ_1a_ subunit is not important for DHPR–RyR1 interaction that underlies skeletal muscle EC coupling.

**Fig. 7.**
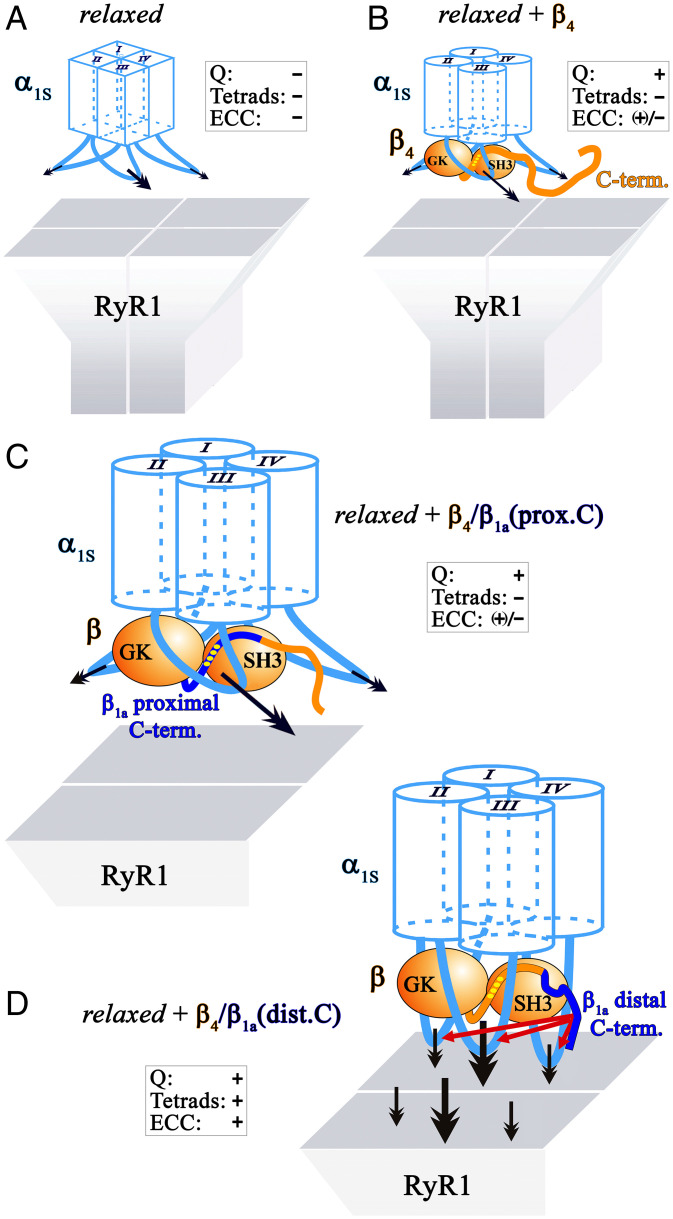
Model of conformational modification of α_1S_ by the β_1a_ distal C terminus—prerequisite for proper skeletal muscle EC coupling. (*A*) In zebrafish mutant *relaxed* due to the absence of the DHPRβ_1a_ subunit, the α_1S_ subunit is in a distorted conformation. This causes impediment of charge movement (*Q*) and of arrangement of DHPR into tetrads (tetrads) that accounts for the lack of skeletal muscle EC coupling (ECC). The distorted conformation of the membrane spanning hydrophobic core regions of the four homologous α_1S_ repeats (I–IV) is depicted by rectangular boxes. The primary and unspecified numbers of secondary α_1S_-specific RyR1 interaction sites (32) are indicated with bold and normal black arrows, respectively. (*B*) β_4_ is unable to reinstate full EC coupling [(+)/−] due to impaired DHPR tetrad formation. According to our model, β_4_ (symbolized in orange) induces proper conformation of the hydrophobic α_1S_ core regions (depicted with cylinders) required for charge movement function, but is unable to reconstitute accurate conformation of the intracellular α_1S_ loops facilitating RyR1 anchoring (tetrad formation). Improper DHPR–RyR1 interaction (tilted arrows) leads to weak EC coupling and impaired tetrad formation. (*C*) Likewise, chimera β_4_/β_1a_(prox.C) in which the proximal C terminus of β_4_ is swapped with corresponding β_1a_ sequence (blue), was unable to reinstate intact tetrad formation and thus full ECC. Yellow dots on the proximal C terminus of the β-subunit depict the intramolecular SH3–PXXP interaction sites critical for charge movement function (16). (*D*) However, the distal C terminus of β_1a_ (blue) enables proper conformation of the intracellular α_1S_ loops crucial for RyR1 anchoring (tetrad formation). Consequently, EC coupling is highly restored upon expression of chimera β_4_/β_1a_(dist.C). The direct DHPR–RyR1 interaction depicted in the model is still obscure. However, it is irrelevant for our conclusions whether the two channels interact directly or via an intermediate protein.

Subsuming all our previous (15, 16) and current observations of the role of β-subunits in functional skeletal muscle DHPR expression, we postulate a molecular model of conformational modifications of DHPRα_1S_ by β-subunits (Fig. 7). In normal muscle cells at rest, DHPRα_1S_ appears to be anchored strongly to RyR1, which results in the arrangement of DHPRα_1S_ in tetrads aligned with RyR1 homotetramers. Functionally, this anchoring is a necessary precondition for coupling depolarization-driven conformational changes of DHPRα_1S_ to the activation of RyR1. In the *relaxed* (β_1_-null) muscle cell, both the membrane-embedded hydrophobic core of α_1S_ and its cytoplasmic domains have nonfunctional conformations so that there is neither charge movement nor tetrad formation, respectively (Fig. 7*A*) and a complete lack of EC coupling. Expression of the β_4_ isoform facilitates a conformation of DHPRα_1S_, which is distinct from the completely nonfunctional conformation in the *relaxed* system (Fig. 7 *A* and *B*). In particular, upon expression of the β_4_ isoform (Fig. 7*B*), domain cooperativity between the SH3 and the PXXP motif in the proximal C terminus induces steric rectification of the hydrophobic core region, enabling the voltage sensing/charge movement function (16). Nevertheless, β_4_ expression is not sufficient to promote accurate conformational restoration of the intracellular regions (loops and C and N terminus) of the α_1S_ subunit (Fig. 7*B*) and consequently is unable to restore full interaction of the DHPR complex with RyR1 resulting in greatly reduced Ca^2+^ transients and impaired tetrad formation. We observed similar behavior for the construct β_4_/β_1a_(prox.C) in which the proximal C terminus of β_4_ is replaced with β_1a_ sequence (Fig. 7*C*). However, some anchoring of α_1S_ to RyR1 must occur for both β_4_ and β_4_/β_1a_(prox.C) because both these β-constructs supported depolarization-induced calcium transients, although attaining peak levels that were only ∼40% of those for β_1a_. A reasonable explanation for the reduced size of these transients is that the anchoring of α_1S_ to RyR1 is weaker for β_4_ and β_4_/β_1a_(prox.C), which would also explain why these constructs did not result in tetradic arrays of α_1S_. In particular, if the probability of α_1S_ binding to one subunit of RyR1 were 40% relative to β_1a_, then the probability of three- and four-particle tetrads would be only 6.4% and 2.6%, respectively.

In stark contrast to the proximal β_1a_ C terminus, the distal C terminus of β_1a_ in chimera β_4_/β_1a_(dist.C) enables appropriate tertiary conformation of the β-subunit (Fig. 7*D*), apt for induction of an accurate conformation of the intracellular molecular regions (loops and termini) of the α_1S_ subunit. This conformational correction finally enables accurate anchoring of the DHPR to RyR1, allowing proper DHPR tetrad formation in orthogonal arrays strictly adjacent to every other RyR1 homotetramer—a key structural basis for full skeletal muscle EC coupling.

Since our results (Fig. 6) indicate that the previously proposed hydrophobic surface motif (L_496_L_500_W_503_) in the distal β_1a_ C terminus (21) is not important for skeletal muscle EC coupling, the question remains as to which of the 35 residues of the distal β_1a_ C terminus are most directly involved for restoring interactions with RyR1. An alternative interaction motif to LLW might be formed by the first 10 residues of the distal C terminus, highly homologous in different β_1a_ distal C termini from fish to mammals (*SI Appendix*, Fig. S2). However, several motif search routines on various sequence databases did not yield promising motif predictions that would justify a targeted alanine replacement strategy on the β_1a_ distal C terminus. Particularly, we could not identify encouraging sequence homologies or motif identities in the C-terminal regions of β_1a_ and β_3_, the only β-subunit beside β_1a_ that also promotes tetrad formation (*SI Appendix*, Fig. S1).

As mentioned above, one could also postulate that a specific secondary structure, adopted only by the distal β_1a_ C terminus might induce the conformation of the intracellular molecular regions (loops and N and C terminus) of the DHPRα_1S_ subunit required for accurate anchoring of the DHPR onto RyR1 (Fig. 7*D*). However, an algorithm that predicts the structure of isolated peptides (28) yielded structures that were roughly similar for the distal C terminus of β_1a_ and the corresponding region of β_4_ despite the low amino acid sequence homology (*SI Appendix*, Fig. S3). Within the full-length proteins, AlphaFold2 predicts that β_1a_ residues V_490_ to L_500_ are alpha helical, whereas β_1a_ residues S_501_ to M_524_ are unstructured, as are all the corresponding residues (S_434_ to K_468_) of β_4_ (β_1a_: https://alphafold.ebi.ac.uk/entry/P19517; β_4_: https://alphafold.ebi.ac.uk/entry/D4A055), but the confidence of the predictions for both β_1a_ and β_4_ ranges from low to very low.

In summary, we found in this study that the heterologous distal C terminus of β_1a_ (amino acid residues V_490_ to M_524_) is critical both for arrangement of DHPRs into tetradic arrays and for full restoration of EC coupling Ca^2+^ release. We could exclude a proposed motif, consisting of the three amino acids L_496_L_500_W_503_ (21) as relevant for accurate DHPR–RyR1 interaction and thus, tetrad formation. Because the currently available alignment and predictive methods did not identify a specific motif or structure, future studies with an allover alanine scan of the distal C terminus of β_1a_ may be necessary for identifying the motif(s)/structure(s) responsible for the key structural prerequisites for EC coupling—DHPR tetrad formation.

## Materials and Methods

### Zebrafish Care.

Breeding and maintenance of adult zebrafish, WT, and heterozygous for the DHPRβ_1_-null mutation *relaxed* (*red*^ts25^) (11) were performed according to established protocols (29, 30). One-day-old postfertilization homozygous *relaxed* zebrafish were recognized by their inability to move in response to tactile stimulation. Motile, heterozygous, and WT siblings, termed “normal” were used as controls. All experimental procedures were approved by the Tierethik-Beirat of the Medical University of Innsbruck and Bundesministerium für Bildung, Wissenschaft und Forschung (BMBWF-66.011/0140-V/3b/2019).

### Expression Plasmids.

Detailed cloning strategies for generation of GFP-tagged cDNAs of β-subunits, chimeras, and mutants are described in SI Appendix, *SI Materials and Methods*.

### Primary Culture of Myotubes.

Myoblasts from 1-dpf *relaxed* zebrafish were isolated, transfected with 2 µg of plasmid cDNA using the Rat Cardiomyocyte Neonatal Nucleofector Kit (Lonza) and cultured in L-15 medium supplemented with 3% fetal calf serum, 3% horse serum, 4 mM L-glutamine, and 4 U/mL penicillin/streptomycin for 4 to 6 d in a humidified incubator at 28.5 °C (30).

### Whole-Cell Patch-Clamp Electrophysiology.

Recordings of intramembrane charge movement as a measure of functional DHPRα_1S_ membrane expression simultaneously with cytoplasmic Ca^2+^ transients were performed on transfected GFP-positive myotubes as previously described (30). Borosilicate glass patch pipettes had a resistance of 3.5 to 5 MΩ when filled with internal solution containing (in millimolar): 100 Cs-aspartate, 10 Hepes, 0.5 Cs-ethylene glycol-bis(-aminoethyl ether)-*N*,*N*,*N'*,*N'*-tetracetic acid, 3 Mg-ATP, and 0.2 Fluo-4 (pH 7.4 with CsOH). *N*-benzyl-p-toluene sulphonamide, Myosin-II blocker (100 μM) was continuously present in the bath (external) solution containing (in millimoles): 10 Ca(OH)_2_, 100 L-aspartate, and 10 Hepes (pH 7.4 with tetraethylammonium hydroxide). All recordings were performed at room temperature (RT).

### mRNA Injection and Freeze-Fracture Electron Microscopy.

Freshly spawned zebrafish embryos were microinjected with in vitro synthesized RNA of GFP-tagged β-subunits, chimeras, or mutants and raised at 28 °C. At 27- to 30-hpf, tails of GFP-positive homozygous *relaxed* zebrafish were fixed in 9% glutaraldehyde in 0.1 M cacodylate buffer (pH 7.2) for 30 min at RT and preserved in 4.5% glutaraldehyde at 4 °C. Tails were mechanically skinned, infiltrated in 30% glycerol in water, fractured in double replica holders, and shadowed with platinum at an angle of 45°, followed by replication with carbon, in a freeze-fracture unit (BFA 400, Balzers S.P.A.) (15). The replicas were examined at the electron microscopy facility of the University of Colorado, Anschutz Medical Campus, using a Tecnai FEI TF20 electron microscope.

### Zebrafish Motility Analysis.

At 17 hpf, normal zebrafish exhibit slow, spontaneous coiling movements and by 21 hpf, multiple coils of the body in response to tactile stimulation can be observed (31). GFP-positive *relaxed* zebrafish, 27- to 30-hpf, injected with β-subunits, chimeras, or mutants were dechorionated using pronase and spontaneous or touch-evoked motility was visually evaluated and degrees of motility were judged according to an assigned scheme (*SI Appendix*, Table S1). Identification and confirmation of the rescued homozygous *relaxed* zebrafish were performed via restriction fragment length polymorphism (RFLP) test (30).

### Statistical Analysis.

Data were analyzed using ClampFit (v10.7, Axon Instruments) and SigmaPlot (v11.0, Systat software, Inc.). Results are expressed as mean ± SEM and *n* = number of myotubes or individual zebrafish. Statistical significance was calculated using unpaired Student’s *t* test and *P* values were set as follows: *P < 0.05, **P < 0.01, and ***P < 0.001.

## Supplementary Material

Supplementary File

## Data Availability

All study data are included in the article and/or *SI Appendix*.
