# Supplementary material for: The distal C terminus of the dihydropyridine receptor β1a subunit is essential for tetrad formation in skeletal muscle
Source: Proc Natl Acad Sci U S A. 2022 May 4;119(19):e2201136119. doi: 10.1073/pnas.2201136119 (PMC9171810; doi:10.1073/pnas.2201136119)
Supplement: Supplementary File [file pnas.2201136119.sapp.pdf]

PNAS

Supplementary Information for

The distal C-terminus of the dihydropyridine receptor β_{1a} subunit is essential for tetrad formation in skeletal muscle

Anamika Dayal[a], Stefano Perni[b], Clara Franzini-Armstrong[c], Kurt G. Beam[b], and Manfred Grabner[a]

[a]*Department of Pharmacology, Medical University of Innsbruck, Peter Mayr Strasse 1A, A-6020, Innsbruck, Austria,* [b]*Department of Physiology and Biophysics, Anschutz Medical Campus, University of Colorado, Aurora, CO 80045,* [c]*Department of Cell and Developmental Biology, University of Pennsylvania School of Medicine, Philadelphia, PA 19104.*

Email: anamika.dayal@i-med.ac.at

This PDF file includes:

Supplementary text
Figures S1 to S3
Tables S1 and S2
SI References

SI Materials and Methods

Generation of N-terminally GFP-tagged cDNAs of β subunits, chimeras, and mutants in expression vector pCI-neo (Promega) is briefly summarized below. Restriction enzyme (RE) sites introduced by PCR are marked with asterisks. Sequence integrity of all expression plasmids was confirmed by sequencing (Eurofins Genomics).

The cloning of GFP-tagged β_{1a} (rabbit; GenBank accession no. M25514) and β_4 (rat; GenBank accession no. L02315) into expression vector pCI-neo has been described previously (1).

β_{1a}/β_4(N). This chimera consists of β_4 N-terminus in β_{1a} sequence background. The β_4/β_{1a} transition site (nucleotide β_4 235 / β_{1a} 262) with β_4 as upstream and β_{1a} as downstream cDNA template was generated using Fusion-PCR. To obtain the final construct β_{1a}/β_4(N), the resulting fusion product AflIII–BstXI (nucleotides β_4 -404 to β_{1a} 835) was co-ligated with the BstXI–XbaI fragment of β_{1a} (nucleotides 835 – 1802) into the corresponding AflIII/XbaI sites (nucleotide -413 / 1802) of the β_{1a} clone.

β_{1a}/β_4(SH3). In this chimera, the conserved Src homology 3 (SH3) domain of β_{1a} was exchanged with the SH3 domain of β_4. Three-fragment fusion PCR was used to generate the β_{1a}/β_4 (nucleotide β_{1a}262 / β_4 235) and β_4/β_{1a} (nucleotide β_4 457 / β_{1a} 484) transition sites. The resultant fusion fragment excised with AflIII and SacI (nucleotides β_{1a} -412 to 696) was co-ligated with SacI–XbaI fragment of β_{1a} (nucleotides 696 – 1802) into the corresponding AflIII/XbaI sites (nucleotide -413 / 1802) of the clone β_{1a} to acquire the final construct β_{1a}/β_4(SH3).

β_{1a}/β_4(H). The variable HOOK (H) region of β_{1a} was replaced with that of β_4. The β_{1a}/β_4 (nucleotide β_{1a} 484 / β_4 457) and β_4/β_{1a} (nucleotide β_4 628 / β_{1a} 796) transition sites were created using three-fragment fusion PCR. To obtain the final construct β_{1a}/β_4(H), the resultant fusion fragment AflIII–BstXI (nucleotides β_{1a} -413 to 835) was co-ligated with BstXI–XbaI fragment of β_{1a} (nucleotides 835 – 1802) into AflIII/XbaI (nucleotides -413 / 1802) of the cleaved β_{1a} clone.

β_{1a}/β_4(GK). This chimera is composed of β_{1a} sequence except for the conserved GK domain, which is derived from β_4. Three-fragment fusion PCR was used to create the β_{1a}/β_4 (nucleotide β_{1a} 796 / β_4 628) and β_4/β_{1a} (nucleotide β_4 1198/ β_{1a} 1366) transition sites. The final fusion fragment SacI–XbaI (nucleotides β_{1a} 696– 1801), along with the AflIII–SacI fragment of β_{1a} (nucleotides -413 to 696) was co-ligated into the corresponding AflIII/XbaI (nucleotide -413 / 1802) sites of the β_{1a} clone to acquire the final construct β_{1a}/β_4(GK).

β_{1a}/β_4(C). In this chimera, the variable C-terminus of β_{1a} is exchanged with that of β_4. The β_{1a}/β_4 transition site (nucleotide β_{1a} 1366/β_4 1198) was introduced by fusion-PCR with β_{1a} as the upstream and β_4 as the downstream cDNA template. For the final construct β_{1a}/β_4(C), the fusion product BstXI–XbaI (nucleotides β_{1a} 835– β_4 1788) was co-ligated with the AflIII–BstXI fragment of β_{1a} (nucleotides -413 to 835) into the corresponding AflIII/XbaI sites (nucleotide -413 / 1802) of clone β_{1a}.

β_4(Δ51). In this truncation mutant, the last 51 residues of the β_4 C-terminus were deleted to obtain identical length of the C-terminus alike β_{1a}. Deletion of nucleotides 1405 – 1562 and introduction of a premature stop (nucleotide 1405) was achieved using fusion PCR with β_4 cDNA as template. For the final construct β_4(Δ51), the resultant PCR product CfoI–XbaI (nucleotides 982 – 1788) was co-ligated with AflIII–CfoI fragment of β_4 (nucleotides -404 – 982) into the AflIII/XbaI (nucleotide -404 / 1788) opened β_4 construct.

β_4/β_{1a}(C). This chimera consists of the β_{1a} C-terminus in β_4 sequence background. Fusion PCR introduced the β_4/β_{1a} transition site (nucleotides β_4 1198 / β_{1a} 1366) and yielded CfoI–XbaI product (nucleotides β_4 982 – β_{1a} 1802). This fusion fragment, together with AflIII–CfoI fragment of β_4 (nucleotides -404 to 982) was ligated into the AflIII/XbaI (nucleotides -404 / 1788) opened β_4, to attain the final chimera β_4/β_{1a}(C).

β_4/β_{1a}(prox.C). In this chimera, the proximal C-terminus of β_{1a} (residues 459-489) is present in β_4(Δ51) sequence background. The β_4/β_{1a} (nucleotide β_4 1198 / β_{1a} 1366) and β_{1a}/β_4 (nucleotide β_{1a} 1467 / β_4 1299) transition sites were produced by fusion PCR, with β_4/β_{1a}(C) as the upstream and β_4(Δ51) as the downstream cDNA template. To acquire chimera β_4/β_{1a}(prox.C), the final PCR fragment CfoI–XbaI (nucleotides 982 – 1788) and AflII–CfoI fragment of β_4 (nucleotides -404 to 982) were co-ligated into the respective AflIII/XbaI (nucleotide -404 / 1788) cleaved β_4 clone.

β_4/β_{1a}(dist.C). This chimera consists of the distal C-terminus of β_{1a} (residues490-524) in β_4(Δ51) sequence background. Fusion PCR was used to create the β_4/β_{1a} (nucleotide β_4 1300 / β_{1a} 1468) transition site. For the final construct β_4/β_{1a}(dist.C), the resultant PCR product CfoI–XbaI (nucleotides β_4 982 – β_{1a} 1802) was co-ligated with AflIII–CfoI fragment of β_4 (nucleotides -404 to 982) into the AflIII/XbaI (nucleotide -404 / 1788) opened β_4 construct.

β_{1a}(LLW-AAA). The LLW/AAA substitutions (L496A, L500A, W503A) were created by using fusion PCR with β_{1a} cDNA as template. Fusion primers for exchange of codons CTC/GCC (nucleotide 1486 – 1488), CTC/GCT (nucleotide 1498 – 1500), and TGG/GCG (nucleotide 1507

– 1509), now coding for alanines instead of leucines and tryptophan were used. Substitution of CTC/GCT (L500A) led to introduction of NheI* site (nucleotide 1499). To gain the final construct, the fusion fragment BstXI–XbaI (nucleotides 835 – 1802) was co-ligated with fragment EcoRV–BstXI of β_{1a} (nucleotides -413 to 835) into the corresponding EcoRV/XbaI (nucleotide -413 / 1802) cleaved β_{1a} clone.

SI Figures

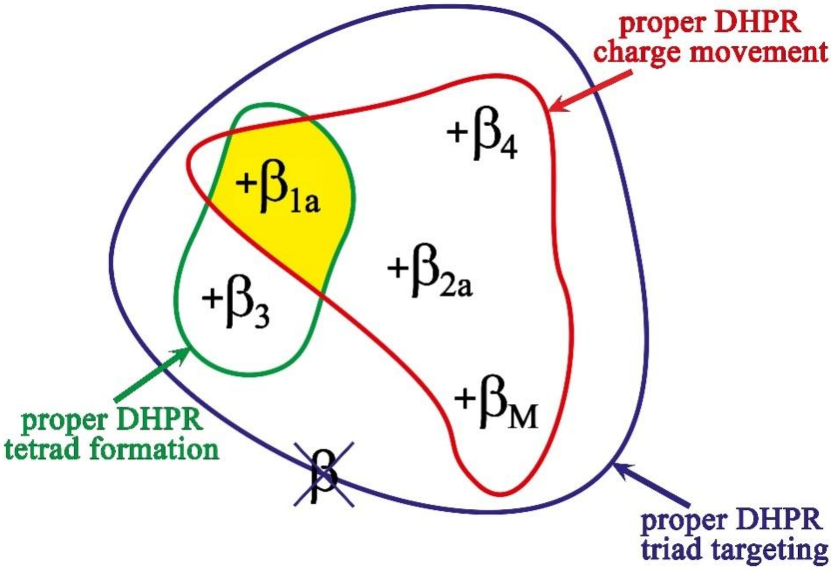

Fig. S1. The three structural-functional prerequisites for proper skeletal-muscle DHPR functioning are promoted to different extents by distinct β isoforms. First the DHPR must be targeted into the triadic membrane of the muscle cell. This basic requirement is fulfilled by expression of all β isoforms (*blue boundary*) investigated so far (2, 3), and strikingly, to a certain extent appears even in β_1-null (*relaxed*) myotubes (β̸) (4). Second, for the canonical DHPR function of perceiving membrane-depolarizations and translating them into a conformational change, DHPR voltage sensing (charge movement) has to be restored accurately. Full DHPR charge movement is promoted by all β isoforms, except β_3 (*red boundary*). Another crucial structural prerequisite for physical DHPR-RyR1 coupling, beside triad targeting, is DHPR tetrad formation, which is the arrangement of DHPRs in groups of four, juxtaposed to every other RyR1 homotetramer. Proper tetrad formation is promoted only by β_{1a} and β_3 (*green boundary*). However, only β_{1a} fulfills all the three structural-functional prerequisites for proper DHPR-RyR1 coupling (*overlay highlighted in yellow*). Consequently, skeletal muscle EC coupling functions fully only upon expression of β_{1a} i.e., 100% intact SR Ca^{2+} release, which results in proper muscle contraction and hence, motility.

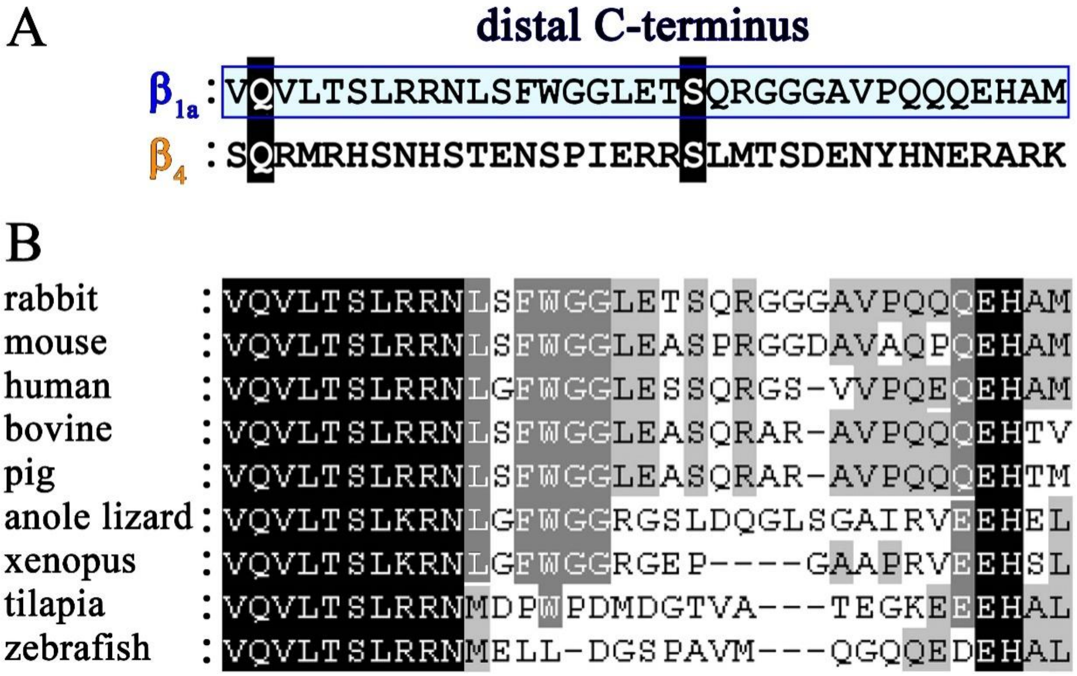

Fig. S2. Amino acid sequence alignments of corresponding segments of distal C-termini of β isoforms. (*A*) Sequence alignment of the distal C-terminus of rabbit β_{1a} (V_{490} - M_{524}) and the corresponding region of rat β_4 (S_{434} - K_{468}) shows a very low homology of 6% between the two isoforms. (*B*) Sequence alignments of distal β_{1a} C-termini from several vertebrate species (fish to mammals) revealed a highly homologous first third followed by a heterologous central- and weaker homologous terminal part. GenBank accession numbers or National Center for Biotechnology Information Gene ID for all β_{1a} sequences aligned are as follows: rabbit (*Oryctolagus cuniculus*), NM_001082279; mouse (*Mus musculus*), NM_031173; human (*Homo sapiens*), NM_000723; bovine (*Bos taurus*), 327703; pig (*Sus scrofa*), XM_003131507; anole lizard (*Anolis carolinensis*), 100564526; Xenopus (*Xenopus tropicalis*), BC154972; tilapia (*Oreochromis niloticus*), XM_003442011; and zebrafish (*Danio rerio*), AY952462.

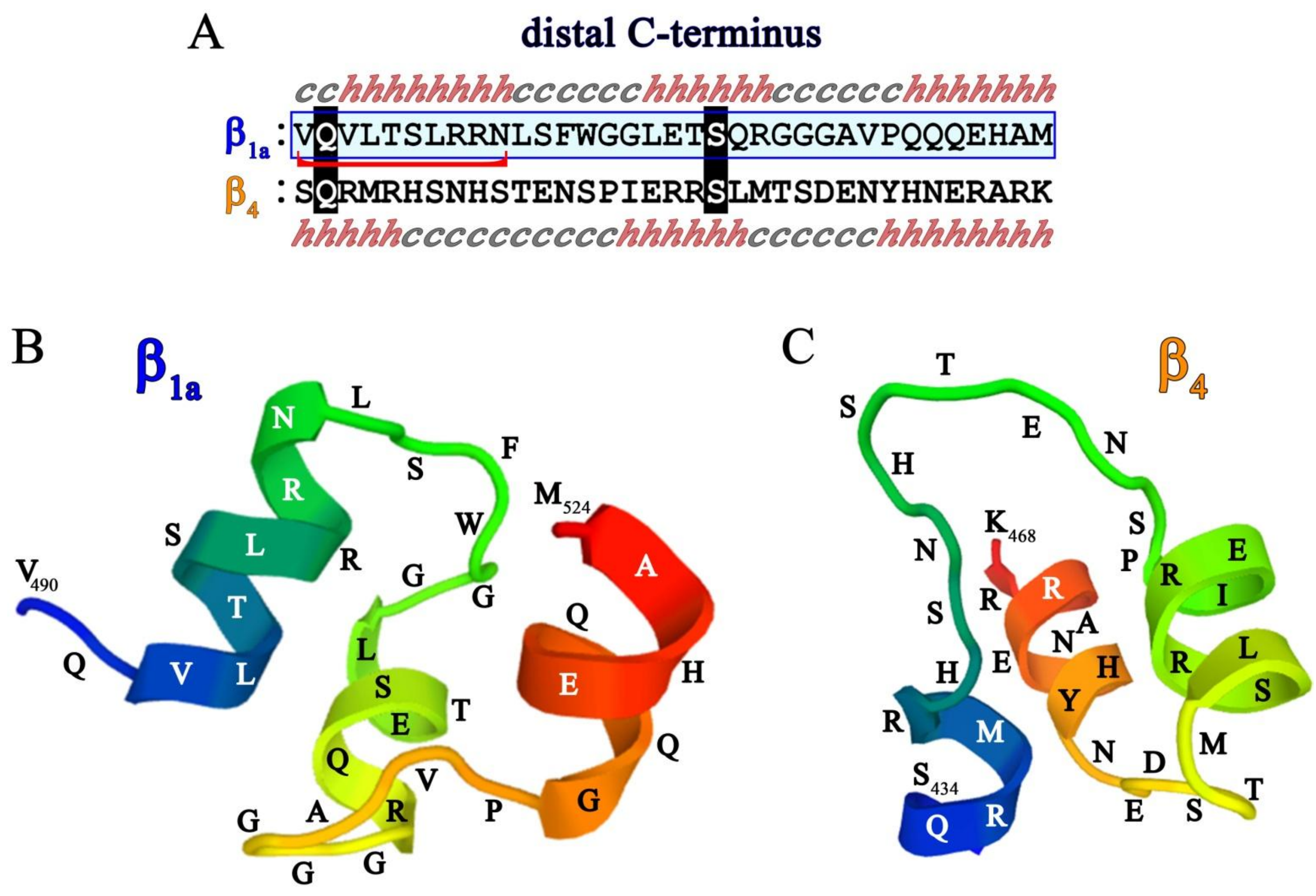

Fig. S3. Similarities in the secondary structures of the distal C-termini of β_{1a} and β_4 isoforms despite high dissimilarities in the primary structures. (*A*) Secondary structure prediction (PEP-FOLD 3.5) displays a striking comparability between the distal C-termini of β_{1a} and $\beta_4$with three α-helices (***h***) connected by random coils (***c***), despite a very low amino acid sequence homology of 6%. Red bracket indicates the highly homologous sequence in the distal C-terminus of β_{1a} revealed from sequence alignments of β_{1a} from several vertebrate species (fish to mammals) (*SI Appendix*, Fig. S2*B*). (*B*) De novo conformation predictions of the distal C-terminus of β_{1a} (V_{490} - M_{524}) and (*C*) β_4 (S_{434} - K_{468}) using the program PEP-FOLD 3.5 (5) on the RPBS web portal. Resulting clusters from 200 independent simulations were sorted by sOPEP energy (6) to yield the "best model" prediction, graphically presented in *cartoon* input style. Positions of amino acids are indicated in one letter code.

Table S1

Spontaneous or touch-evoked motility of 27-30 hpf GFP-positive *relaxed* zebrafish injected with different β constructs was visually evaluated and degree of motility was judged according to the assigned scheme. + signs were translated into numerical values for statistical processing of the data as mean ± SEM.

**degree of motility**		**assigned numerical value**
**normal**	**++++**	**4**
**slightly weaker than normal**	**+++**	**3**
**weak**	**++**	**2**
**very weak**	**+**	**1**
**no motility / *relaxed***	**-**	**0**

Table S2

Numbers of tetrads (three- or four-particles) counted by two independent investigators from anonymized freeze-fracture images.

Investigator		untransfected		*relaxed* transfected		
	Tetrads	*relaxed*	normal	β_4	β_4/β_{1a}(C)	β_4/β_{1a}(dist.C)
#1	Three particles	7	21	1	23	23
	Four particles	3	34	0	29	10
#2	Three particles	0	37	0	27	13
	Four particles	0	30	1	25	6

SI References

1. A. Dayal, V. Bhat, C. Franzini-Armstrong, M. Grabner, Domain cooperativity in the β_{1a} subunit is essential for dihydropyridine receptor voltage sensing in skeletal muscle. *Proc. Natl. Acad. Sci. U.S.A.* **110(18)**, 7488-7493 (2013).
2. J. Schredelseker, A. Dayal, T. Schwerte, C. Franzini-Armstrong, M. Grabner, Proper restoration of excitation-contraction coupling in the dihydropyridine receptor β_1-null zebrafish relaxed is an exclusive function of the β_{1a} subunit. *J. Biol. Chem.* **284(2)**, 1242-1251 (2009).
3. B. A. Simms, G. W. Zamponi, Trafficking and stability of voltage-gated calcium channels. *Cell. Mol. Life Sci.* **69(6),** 843-856 (2012).
4. J. Schredelseker et al., The β_{1a} subunit is essential for the assembly of dihydropyridine-receptor arrays in skeletal muscle. *Proc. Natl. Acad. Sci. U.S.A.* **102(47)**, 17219-17224 (2005).
5. P. Thévenet et al., PEP-FOLD: an updated de novo structure prediction server for both linear and disulfide bonded cyclic peptides. *Nucleic Acids Res.*, **40** (Web Server issue), W288-W293 (2012).
6. Z. Wang et al., APOLLO: a quality assessment service for single and multiple protein models. *Bioinformatics*, **27,** 1715-1716 (2011).
